# *N*,*N*′-Bis(2-chloro­benz­yl)-*N*′′-(2,2,2-trichloro­acet­yl)phospho­ric triamide

**DOI:** 10.1107/S1600536811027681

**Published:** 2011-07-16

**Authors:** Mehrdad Pourayoubi, Hassan Fadaei, Masood Parvez

**Affiliations:** a Department of Chemistry, Ferdowsi University of Mashhad, Mashhad 91779, Iran; b Department of Chemistry, University of Calgary, 2500 University Drive N.W., Calgary, Alberta, Canada T2N 1N4

## Abstract

The P atom in the title compound, C_16_H_15_Cl_5_N_3_O_2_P, exhibits a tetra­hedral coordination geometry and the phosphoryl and carbonyl groups are *anti* with respect to one another. The dihedral angle between the benzene rings is 44.90 (15)°. One of the 2-chloro­benzyl­amido fragments is disordered over two sets of sites with occupancies of 0.8823 (17) and 0.1177 (17). In the crystal, adjacent mol­ecules are linked *via* N—H⋯O(P) and N—H⋯O(C) hydrogen bonds into an extended chain running parallel to the *a* axis.

## Related literature

For details of compounds having a C(O)NHP(O) skeleton, see: Toghraee *et al.* (2011 ▶). For bond lengths in related structures, see: Pourayoubi *et al.* (2011 ▶); Rudd *et al.* (1996 ▶).
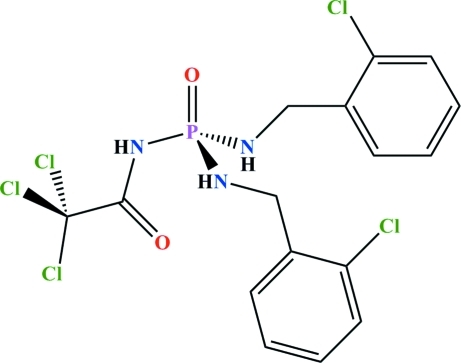


## Experimental

### 

#### Crystal data

C_16_H_15_Cl_5_N_3_O_2_P
*M* *_r_* = 489.53
Triclinic, 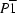

*a* = 9.9789 (2) Å
*b* = 10.6058 (3) Å
*c* = 10.8386 (3) Å
α = 75.8920 (13)°
β = 72.2250 (15)°
γ = 69.6050 (15)°
*V* = 1011.66 (4) Å^3^
*Z* = 2
Mo *K*α radiation
μ = 0.81 mm^−1^
*T* = 173 K
0.18 × 0.14 × 0.12 mm

#### Data collection

Nonius KappaCCD diffractometer with APEXII CCD detector
Absorption correction: multi-scan (*SORTAV*; Blessing, 1997 ▶) *T*_min_ = 0.867, *T*_max_ = 0.909
8947 measured reflections
4638 independent reflections
4083 reflections with *I* > 2σ(*I*)
*R*_int_ = 0.020

#### Refinement

*R*[*F* ^2^ > 2σ(*F* ^2^)] = 0.053
*wR*(*F* ^2^) = 0.129
*S* = 1.05
4638 reflections
245 parameters
H-atom parameters constrained
Δρ_max_ = 1.42 e Å^−3^
Δρ_min_ = −0.68 e Å^−3^

### 

Data collection: *COLLECT* (Hooft, 1998 ▶); cell refinement: *DENZO* (Otwinowski & Minor, 1997 ▶); data reduction: *SCALEPACK* (Otwinowski & Minor, 1997 ▶); program(s) used to solve structure: *SIR92* (Altomare *et al.*, 1993 ▶); program(s) used to refine structure: *SHELXL97* (Sheldrick, 2008 ▶); molecular graphics: *Mercury* (Macrae *et al.*, 2008 ▶); software used to prepare material for publication: *enCIFer* (Allen *et al.*, 2004 ▶).

## Supplementary Material

Crystal structure: contains datablock(s) I, global. DOI: 10.1107/S1600536811027681/sj5176sup1.cif

Structure factors: contains datablock(s) I. DOI: 10.1107/S1600536811027681/sj5176Isup2.hkl

Additional supplementary materials:  crystallographic information; 3D view; checkCIF report

## Figures and Tables

**Table 1:** Hydrogen-bond geometry (Å, °)

*D*—H⋯*A*	*D*—H	H⋯*A*	*D*⋯*A*	*D*—H⋯*A*
N1—H1⋯O2^i^	0.88	1.94	2.804 (3)	168
N2—H2⋯O1^ii^	0.88	2.38	3.106 (3)	141
N2′—H2′⋯O1	0.88	2.42	3.025 (3)	126
N3—H3⋯O1^ii^	0.88	2.34	3.129 (3)	149

Symmetry codes: (i) 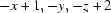; (ii) 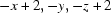.

**Fig. 1.**
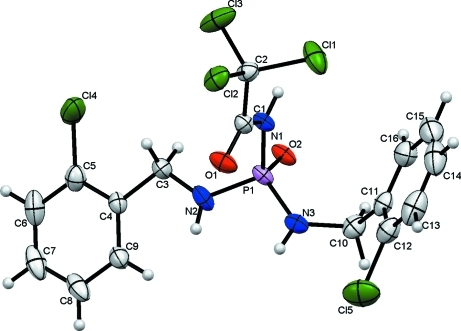
The molecular structure of the title compound with ellipsoids shown at the 50% probability level. The minor disorder component is not shown.

## supplementary crystallographic information

### Comment

The structure determination of the title compound, P(O)[NHC(O)CCl_3_][NHCH_2_C_6_H_4_(2-Cl)]_2_ (Fig. 1), was performed as a part of a project in our laboratory on the synthesis of new phosphoramidate compounds having a C(O)NHP(O) skeleton (Toghraee *et al.*, 2011). Single crystals of the title compound were obtained from a solution of CH_3_OH after slow evaporation at room temperature.

The P═O (1.472 (2) Å) and C═O (1.212 (3) Å) bond lengths are standard for this category of compounds (Pourayoubi *et al.*, 2011). The P atom has a distorted tetrahedral configuration (Fig. 1) as has been noted for other phosphoric triamides and their chalco-derivatives (Rudd *et al.*, 1996). The bond angles at the P atom vary in the range  103.26 (13)- 116.10 (12)°. The P—N2 and P—N3 bonds (with lengths of 1.621 (2) Å and 1.622 (2) Å) are shorter than the P—N1 bond (1.715 (2) Å). As can be expected the C1—N1 bond distance (1.339 (3) Å) is shorter than the other C—N bond distances. The N—H unit of C(O)NHP(O) moiety and the phosphoryl group have a *syn* orientation with respect to each other. One of the 2-chlorobenzylamido fragments is disordered over two sites with occupancies of 0.8823 (17) and 0.1177 (17).

In the crystal, each molecule is hydrogen-bonded to two adjacent molecules through N_C(O)NHP(O)_—H···O(P) and N—H···O(C) hydrogen bonds forming linear chains parallel to [100] (Table 1).

### Experimental

The reaction of phosphorus pentachloride (35.6 mmol) and 2,2,2-trichloroacetamide (35.6 mmol) in dry CCl_4_ (25 ml) at 353 K (3 h) and then treatment with formic acid 85% (35.6 mmol) at 271 K leads to the formation of CCl_3_C(O)NHP(O)Cl_2_ as a white solid.

To a solution of CCl_3_C(O)NHP(O)Cl_2_ (2.5 mmol) in dry CHCl_3_ (30 ml), a solution of 2-chlorobenzylamine (10 mmol) in dry CHCl_3_ (10 ml) was added dropwise at 271 K. After 4 h stirring, the solvent was evaporated at room temperature. The solid was washed with distilled water and recrystallized from CH_3_OH.

### Refinement

The H-atoms were included at geometrically idealized positions with distances N—H = 0.88 Å and C—H = 0.95 and 0.99 Å for aryl and methylene type H-atoms, respectively. The chlorobenzyl group attached to N2 was disordered with its atoms located over two sites with site occupancy factors 0.8823 (17) and 0.1177 (17). The H-atoms were assigned *U*_iso_ = 1.2 times *U*_eq_ of the parent atoms (C/N). The highest electron density peak in the final difference map was located close to a Cl atom and was essentially meaningless.

### Crystal data

**Table:**

C_16_H_15_Cl_5_N_3_O_2_P	*Z* = 2
*M**_r_* = 489.53	*F*(000) = 496
Triclinic, *P*1	*D*_x_ = 1.607 Mg m^−^^3^
Hall symbol: -P 1	Mo *K*α radiation, λ = 0.71073 Å
*a* = 9.9789 (2) Å	Cell parameters from 4567 reflections
*b* = 10.6058 (3) Å	θ = 1.0–27.5°
*c* = 10.8386 (3) Å	µ = 0.81 mm^−^^1^
α = 75.8920 (13)°	*T* = 173 K
β = 72.2250 (15)°	Prism, colorless
γ = 69.6050 (15)°	0.18 × 0.14 × 0.12 mm
*V* = 1011.66 (4) Å^3^	

### Data collection

**Table:**

Nonius KappaCCD diffractometer with APEXII CCD detector	4638 independent reflections
Radiation source: fine-focus sealed tube	4083 reflections with *I* > 2σ(*I*)
graphite	*R*_int_ = 0.020
ω and φ scans	θ_max_ = 27.6°, θ_min_ = 2.0°
Absorption correction: multi-scan (*SORTAV*; Blessing, 1997)	*h* = −12→12
*T*_min_ = 0.867, *T*_max_ = 0.909	*k* = −13→13
8947 measured reflections	*l* = −14→14

### Refinement

**Table:**

Refinement on *F*^2^	Primary atom site location: structure-invariant direct methods
Least-squares matrix: full	Secondary atom site location: difference Fourier map
*R*[*F*^2^ > 2σ(*F*^2^)] = 0.053	Hydrogen site location: inferred from neighbouring sites
*wR*(*F*^2^) = 0.129	H-atom parameters constrained
*S* = 1.05	*w* = 1/[σ^2^(*F*_o_^2^) + (0.043*P*)^2^ + 2.0234*P*] where *P* = (*F*_o_^2^ + 2*F*_c_^2^)/3
4638 reflections	(Δ/σ)_max_ < 0.001
245 parameters	Δρ_max_ = 1.42 e Å^−^^3^
0 restraints	Δρ_min_ = −0.68 e Å^−^^3^

### Special details

**Table:**

Experimental. IR (KBr, cm^-1^): 3371, 3061, 2875, 1692, 1448, 1257, 1224, 1076, 1043, 885, 837, 751, 675.
Geometry. All e.s.d.'s (except the e.s.d. in the dihedral angle between two l.s. planes) are estimated using the full covariance matrix. The cell e.s.d.'s are taken into account individually in the estimation of e.s.d.'s in distances, angles and torsion angles; correlations between e.s.d.'s in cell parameters are only used when they are defined by crystal symmetry. An approximate (isotropic) treatment of cell e.s.d.'s is used for estimating e.s.d.'s involving l.s. planes.
Refinement. Refinement of *F*^2^ against ALL reflections. The weighted *R*-factor *wR* and goodness of fit *S* are based on *F*^2^, conventional *R*-factors *R* are based on *F*, with *F* set to zero for negative *F*^2^. The threshold expression of *F*^2^ > σ(*F*^2^) is used only for calculating *R*-factors(gt) *etc*. and is not relevant to the choice of reflections for refinement. *R*-factors based on *F*^2^ are statistically about twice as large as those based on *F*, and *R*- factors based on ALL data will be even larger.

### Fractional atomic coordinates and isotropic or equivalent isotropic displacement parameters (Å^2^)

**Table:**

	*x*	*y*	*z*	*U*_iso_*/*U*_eq_	Occ. (<1)
Cl1	0.58102 (9)	−0.04802 (11)	1.36183 (8)	0.0547 (3)	
Cl2	0.87488 (8)	−0.22348 (7)	1.35952 (8)	0.03998 (19)	
Cl3	0.68228 (12)	−0.30242 (9)	1.26210 (11)	0.0590 (3)	
P1	0.72798 (7)	0.04068 (7)	0.90391 (6)	0.02507 (16)	
O1	0.9079 (2)	−0.0722 (2)	1.1030 (2)	0.0386 (5)	
O2	0.60042 (19)	0.0572 (2)	0.85370 (18)	0.0317 (4)	
N1	0.6903 (2)	−0.0446 (2)	1.0605 (2)	0.0262 (4)	
H1	0.6044	−0.0605	1.0925	0.031*	
C1	0.7866 (3)	−0.0882 (3)	1.1360 (3)	0.0259 (5)	
C2	0.7338 (3)	−0.1636 (3)	1.2751 (3)	0.0301 (5)	
N2	0.8865 (2)	−0.0463 (3)	0.8250 (2)	0.0352 (6)	
H2	0.9630	−0.0151	0.8031	0.042*	0.8823 (17)
C3	0.9024 (3)	−0.1740 (3)	0.7909 (3)	0.0335 (7)	0.8823 (17)
H3A	0.8074	−0.1713	0.7779	0.040*	0.8823 (17)
H3B	0.9247	−0.2470	0.8649	0.040*	0.8823 (17)
C4	1.0236 (2)	−0.2094 (2)	0.66667 (18)	0.0269 (7)	0.8823 (17)
C5	1.0496 (2)	−0.3322 (2)	0.6261 (2)	0.0362 (7)	0.8823 (17)
C6	1.1578 (3)	−0.3675 (2)	0.5135 (2)	0.0476 (10)	0.8823 (17)
H6	1.1755	−0.4515	0.4858	0.057*	0.8823 (17)
C7	1.2401 (3)	−0.2800 (3)	0.4415 (2)	0.0501 (11)	0.8823 (17)
H7	1.3141	−0.3041	0.3645	0.060*	0.8823 (17)
C8	1.2141 (2)	−0.1572 (2)	0.4820 (2)	0.0467 (9)	0.8823 (17)
H8	1.2704	−0.0973	0.4328	0.056*	0.8823 (17)
C9	1.1059 (2)	−0.12185 (18)	0.5946 (2)	0.0342 (7)	0.8823 (17)
H9	1.0882	−0.0379	0.6223	0.041*	0.8823 (17)
Cl4	0.94994 (12)	−0.44175 (10)	0.71412 (13)	0.0605 (3)	0.8823 (17)
H2'	0.9162	−0.1096	0.8886	0.042*	0.1177 (17)
C3'	0.993 (3)	−0.079 (2)	0.730 (2)	0.0335 (7)	0.1177 (17)
H3'1	1.0801	−0.0997	0.7645	0.040*	0.1177 (17)
H3'2	0.9892	0.0079	0.6686	0.040*	0.1177 (17)
C4'	1.036 (2)	−0.1806 (18)	0.6432 (18)	0.0269 (7)	0.1177 (17)
C5'	1.148 (2)	−0.1813 (19)	0.529 (2)	0.0362 (7)	0.1177 (17)
C6'	1.199 (2)	−0.289 (2)	0.4586 (18)	0.0476 (10)	0.1177 (17)
H6'	1.2753	−0.2892	0.3808	0.057*	0.1177 (17)
C7'	1.137 (2)	−0.395 (2)	0.501 (2)	0.0501 (11)	0.1177 (17)
H7'	1.1711	−0.4687	0.4529	0.060*	0.1177 (17)
C8'	1.024 (2)	−0.3946 (17)	0.6152 (19)	0.0467 (9)	0.1177 (17)
H8'	0.9819	−0.4675	0.6445	0.056*	0.1177 (17)
C9'	0.9738 (19)	−0.287 (2)	0.6861 (15)	0.0342 (7)	0.1177 (17)
H9'	0.8970	−0.2867	0.7638	0.041*	0.1177 (17)
Cl4'	1.2278 (9)	−0.0556 (8)	0.4817 (10)	0.0605 (3)	0.1177 (17)
N3	0.7555 (2)	0.1823 (2)	0.9050 (2)	0.0309 (5)	
H3	0.8465	0.1849	0.8889	0.037*	
C10	0.6334 (3)	0.3062 (3)	0.9322 (3)	0.0356 (6)	
H10A	0.5478	0.3027	0.9065	0.043*	
H10B	0.6632	0.3860	0.8773	0.043*	
C11	0.5868 (3)	0.3256 (3)	1.0740 (3)	0.0322 (6)	
C12	0.6657 (4)	0.3724 (3)	1.1291 (3)	0.0409 (7)	
C13	0.6224 (5)	0.3866 (4)	1.2614 (4)	0.0529 (9)	
H13	0.6780	0.4188	1.2971	0.064*	
C14	0.4988 (5)	0.3535 (4)	1.3393 (4)	0.0584 (10)	
H14	0.4689	0.3624	1.4296	0.070*	
C15	0.4189 (4)	0.3081 (4)	1.2880 (4)	0.0559 (9)	
H15	0.3331	0.2856	1.3424	0.067*	
C16	0.4620 (4)	0.2945 (3)	1.1571 (3)	0.0437 (7)	
H16	0.4047	0.2629	1.1228	0.052*	
Cl5	0.82320 (12)	0.41376 (12)	1.03083 (12)	0.0672 (3)	

### Atomic displacement parameters (Å^2^)

**Table:**

	*U*^11^	*U*^22^	*U*^33^	*U*^12^	*U*^13^	*U*^23^
Cl1	0.0395 (4)	0.0749 (6)	0.0293 (4)	0.0045 (4)	−0.0024 (3)	−0.0105 (4)
Cl2	0.0424 (4)	0.0350 (4)	0.0463 (4)	−0.0098 (3)	−0.0260 (3)	0.0039 (3)
Cl3	0.0763 (6)	0.0416 (5)	0.0793 (7)	−0.0360 (4)	−0.0472 (5)	0.0180 (4)
P1	0.0178 (3)	0.0380 (4)	0.0204 (3)	−0.0100 (3)	−0.0013 (2)	−0.0080 (3)
O1	0.0246 (10)	0.0590 (14)	0.0358 (11)	−0.0193 (9)	−0.0083 (8)	−0.0023 (10)
O2	0.0199 (9)	0.0517 (12)	0.0251 (9)	−0.0139 (8)	−0.0036 (7)	−0.0063 (8)
N1	0.0195 (10)	0.0363 (12)	0.0235 (10)	−0.0118 (9)	−0.0025 (8)	−0.0042 (9)
C1	0.0224 (12)	0.0282 (13)	0.0269 (13)	−0.0077 (10)	−0.0050 (10)	−0.0049 (10)
C2	0.0302 (13)	0.0271 (13)	0.0333 (14)	−0.0094 (10)	−0.0119 (11)	0.0013 (10)
N2	0.0201 (10)	0.0533 (15)	0.0354 (13)	−0.0106 (10)	0.0012 (9)	−0.0232 (11)
C3	0.0285 (15)	0.0314 (16)	0.0339 (16)	−0.0083 (12)	0.0032 (12)	−0.0078 (13)
C4	0.0245 (13)	0.0277 (16)	0.0247 (15)	−0.0021 (12)	−0.0062 (11)	−0.0053 (12)
C5	0.0303 (16)	0.0358 (17)	0.0444 (19)	−0.0038 (13)	−0.0142 (14)	−0.0117 (14)
C6	0.039 (2)	0.052 (2)	0.050 (2)	0.0103 (17)	−0.0210 (17)	−0.0285 (19)
C7	0.040 (2)	0.068 (3)	0.0244 (17)	0.0100 (19)	−0.0069 (15)	−0.0146 (17)
C8	0.0312 (17)	0.061 (2)	0.0269 (17)	0.0004 (16)	−0.0008 (14)	0.0019 (16)
C9	0.0285 (15)	0.0358 (17)	0.0307 (16)	−0.0046 (13)	−0.0026 (12)	−0.0050 (13)
Cl4	0.0475 (5)	0.0380 (5)	0.0988 (9)	−0.0148 (4)	−0.0086 (5)	−0.0240 (5)
N2'	0.0201 (10)	0.0533 (15)	0.0354 (13)	−0.0106 (10)	0.0012 (9)	−0.0232 (11)
C3'	0.0285 (15)	0.0314 (16)	0.0339 (16)	−0.0083 (12)	0.0032 (12)	−0.0078 (13)
C4'	0.0245 (13)	0.0277 (16)	0.0247 (15)	−0.0021 (12)	−0.0062 (11)	−0.0053 (12)
C5'	0.0303 (16)	0.0358 (17)	0.0444 (19)	−0.0038 (13)	−0.0142 (14)	−0.0117 (14)
C6'	0.039 (2)	0.052 (2)	0.050 (2)	0.0103 (17)	−0.0210 (17)	−0.0285 (19)
C7'	0.040 (2)	0.068 (3)	0.0244 (17)	0.0100 (19)	−0.0069 (15)	−0.0146 (17)
C8'	0.0312 (17)	0.061 (2)	0.0269 (17)	0.0004 (16)	−0.0008 (14)	0.0019 (16)
C9'	0.0285 (15)	0.0358 (17)	0.0307 (16)	−0.0046 (13)	−0.0026 (12)	−0.0050 (13)
Cl4'	0.0475 (5)	0.0380 (5)	0.0988 (9)	−0.0148 (4)	−0.0086 (5)	−0.0240 (5)
N3	0.0251 (11)	0.0398 (13)	0.0293 (12)	−0.0138 (10)	−0.0051 (9)	−0.0035 (10)
C10	0.0399 (15)	0.0336 (15)	0.0342 (15)	−0.0088 (12)	−0.0169 (12)	0.0002 (11)
C11	0.0369 (14)	0.0232 (13)	0.0353 (15)	−0.0020 (11)	−0.0174 (12)	−0.0017 (11)
C12	0.0452 (17)	0.0320 (15)	0.0484 (18)	−0.0072 (13)	−0.0219 (14)	−0.0041 (13)
C13	0.073 (3)	0.0379 (18)	0.054 (2)	0.0001 (16)	−0.038 (2)	−0.0127 (15)
C14	0.063 (2)	0.050 (2)	0.0396 (19)	0.0104 (18)	−0.0097 (17)	−0.0114 (16)
C15	0.048 (2)	0.053 (2)	0.048 (2)	−0.0016 (16)	−0.0025 (16)	−0.0043 (17)
C16	0.0402 (17)	0.0381 (17)	0.0478 (19)	−0.0070 (13)	−0.0101 (14)	−0.0053 (14)
Cl5	0.0632 (6)	0.0785 (7)	0.0774 (7)	−0.0424 (5)	−0.0238 (5)	−0.0030 (5)

### Geometric parameters (Å, °)

**Table:**

Cl1—C2	1.759 (3)	C3'—H3'2	0.9900
Cl2—C2	1.761 (3)	C4'—C5'	1.3900
Cl3—C2	1.768 (3)	C4'—C9'	1.3900
P1—O2	1.4724 (18)	C5'—C6'	1.3900
P1—N2	1.621 (2)	C5'—Cl4'	1.682 (17)
P1—N3	1.622 (2)	C6'—C7'	1.3900
P1—N1	1.715 (2)	C6'—H6'	0.9500
O1—C1	1.212 (3)	C7'—C8'	1.3900
N1—C1	1.339 (3)	C7'—H7'	0.9500
N1—H1	0.8800	C8'—C9'	1.3900
C1—C2	1.553 (4)	C8'—H8'	0.9500
N2—C3	1.434 (4)	C9'—H9'	0.9500
N2—H2	0.8800	N3—C10	1.467 (4)
C3—C4	1.534 (3)	N3—H3	0.8800
C3—H3A	0.9900	C10—C11	1.507 (4)
C3—H3B	0.9900	C10—H10A	0.9900
C4—C5	1.3900	C10—H10B	0.9900
C4—C9	1.3900	C11—C12	1.388 (4)
C5—C6	1.3900	C11—C16	1.389 (4)
C5—Cl4	1.713 (2)	C12—C13	1.395 (5)
C6—C7	1.3900	C12—Cl5	1.740 (4)
C6—H6	0.9500	C13—C14	1.371 (6)
C7—C8	1.3900	C13—H13	0.9500
C7—H7	0.9500	C14—C15	1.359 (6)
C8—C9	1.3900	C14—H14	0.9500
C8—H8	0.9500	C15—C16	1.380 (5)
C9—H9	0.9500	C15—H15	0.9500
C3'—C4'	1.47 (3)	C16—H16	0.9500
C3'—H3'1	0.9900		
			
O2—P1—N2	116.10 (12)	H3'1—C3'—H3'2	105.4
O2—P1—N3	114.51 (12)	C5'—C4'—C9'	120.0
N2—P1—N3	103.26 (13)	C5'—C4'—C3'	121.6 (16)
O2—P1—N1	104.46 (11)	C9'—C4'—C3'	118.0 (16)
N2—P1—N1	108.13 (13)	C4'—C5'—C6'	120.0
N3—P1—N1	110.31 (11)	C4'—C5'—Cl4'	119.8 (12)
C1—N1—P1	122.56 (18)	C6'—C5'—Cl4'	120.1 (12)
C1—N1—H1	118.7	C5'—C6'—C7'	120.0
P1—N1—H1	118.7	C5'—C6'—H6'	120.0
O1—C1—N1	124.8 (2)	C7'—C6'—H6'	120.0
O1—C1—C2	119.8 (2)	C8'—C7'—C6'	120.0
N1—C1—C2	115.5 (2)	C8'—C7'—H7'	120.0
C1—C2—Cl1	108.21 (18)	C6'—C7'—H7'	120.0
C1—C2—Cl2	110.50 (18)	C7'—C8'—C9'	120.0
Cl1—C2—Cl2	109.24 (16)	C7'—C8'—H8'	120.0
C1—C2—Cl3	109.63 (19)	C9'—C8'—H8'	120.0
Cl1—C2—Cl3	109.77 (15)	C8'—C9'—C4'	120.0
Cl2—C2—Cl3	109.47 (15)	C8'—C9'—H9'	120.0
C3—N2—P1	120.0 (2)	C4'—C9'—H9'	120.0
C3—N2—H2	120.0	C10—N3—P1	122.06 (19)
P1—N2—H2	120.0	C10—N3—H3	119.0
N2—C3—C4	113.7 (2)	P1—N3—H3	119.0
N2—C3—H3A	108.8	N3—C10—C11	113.5 (2)
C4—C3—H3A	108.8	N3—C10—H10A	108.9
N2—C3—H3B	108.8	C11—C10—H10A	108.9
C4—C3—H3B	108.8	N3—C10—H10B	108.9
H3A—C3—H3B	107.7	C11—C10—H10B	108.9
C5—C4—C9	120.0	H10A—C10—H10B	107.7
C5—C4—C3	118.28 (18)	C12—C11—C16	116.5 (3)
C9—C4—C3	121.71 (18)	C12—C11—C10	122.8 (3)
C4—C5—C6	120.0	C16—C11—C10	120.7 (3)
C4—C5—Cl4	120.65 (14)	C11—C12—C13	121.7 (3)
C6—C5—Cl4	119.35 (14)	C11—C12—Cl5	119.1 (3)
C7—C6—C5	120.0	C13—C12—Cl5	119.1 (3)
C7—C6—H6	120.0	C14—C13—C12	119.3 (3)
C5—C6—H6	120.0	C14—C13—H13	120.3
C6—C7—C8	120.0	C12—C13—H13	120.3
C6—C7—H7	120.0	C15—C14—C13	120.3 (4)
C8—C7—H7	120.0	C15—C14—H14	119.9
C9—C8—C7	120.0	C13—C14—H14	119.9
C9—C8—H8	120.0	C14—C15—C16	120.1 (4)
C7—C8—H8	120.0	C14—C15—H15	119.9
C8—C9—C4	120.0	C16—C15—H15	119.9
C8—C9—H9	120.0	C15—C16—C11	121.9 (3)
C4—C9—H9	120.0	C15—C16—H16	119.0
C4'—C3'—H3'1	103.9	C11—C16—H16	119.0
C4'—C3'—H3'2	103.9		
			
O2—P1—N1—C1	174.8 (2)	C9'—C4'—C5'—C6'	0.0
N2—P1—N1—C1	50.6 (2)	C3'—C4'—C5'—C6'	172 (2)
N3—P1—N1—C1	−61.6 (2)	C9'—C4'—C5'—Cl4'	−177.3 (15)
P1—N1—C1—O1	0.2 (4)	C3'—C4'—C5'—Cl4'	−5(2)
P1—N1—C1—C2	−179.98 (18)	C4'—C5'—C6'—C7'	0.0
O1—C1—C2—Cl1	116.0 (3)	Cl4'—C5'—C6'—C7'	177.2 (16)
N1—C1—C2—Cl1	−63.9 (3)	C5'—C6'—C7'—C8'	0.0
O1—C1—C2—Cl2	−3.6 (3)	C6'—C7'—C8'—C9'	0.0
N1—C1—C2—Cl2	176.56 (19)	C7'—C8'—C9'—C4'	0.0
O1—C1—C2—Cl3	−124.3 (2)	C5'—C4'—C9'—C8'	0.0
N1—C1—C2—Cl3	55.8 (3)	C3'—C4'—C9'—C8'	−172 (2)
O2—P1—N2—C3	−47.0 (3)	O2—P1—N3—C10	34.9 (2)
N3—P1—N2—C3	−173.2 (2)	N2—P1—N3—C10	162.0 (2)
N1—P1—N2—C3	69.9 (3)	N1—P1—N3—C10	−82.6 (2)
P1—N2—C3—C4	151.5 (2)	P1—N3—C10—C11	94.5 (3)
N2—C3—C4—C5	179.0 (2)	N3—C10—C11—C12	77.3 (3)
N2—C3—C4—C9	−1.5 (4)	N3—C10—C11—C16	−101.5 (3)
C9—C4—C5—C6	0.0	C16—C11—C12—C13	0.4 (4)
C3—C4—C5—C6	179.5 (2)	C10—C11—C12—C13	−178.5 (3)
C9—C4—C5—Cl4	179.67 (18)	C16—C11—C12—Cl5	−179.9 (2)
C3—C4—C5—Cl4	−0.8 (2)	C10—C11—C12—Cl5	1.2 (4)
C4—C5—C6—C7	0.0	C11—C12—C13—C14	0.0 (5)
Cl4—C5—C6—C7	−179.67 (18)	Cl5—C12—C13—C14	−179.7 (3)
C5—C6—C7—C8	0.0	C12—C13—C14—C15	−0.3 (5)
C6—C7—C8—C9	0.0	C13—C14—C15—C16	0.2 (6)
C7—C8—C9—C4	0.0	C14—C15—C16—C11	0.2 (5)
C5—C4—C9—C8	0.0	C12—C11—C16—C15	−0.5 (5)
C3—C4—C9—C8	−179.5 (3)	C10—C11—C16—C15	178.4 (3)

### Hydrogen-bond geometry (Å, °)

**Table:**

*D*—H···*A*	*D*—H	H···*A*	*D*···*A*	*D*—H···*A*
N1—H1···O2^i^	0.88	1.94	2.804 (3)	168.
N2—H2···O1^ii^	0.88	2.38	3.106 (3)	141.
N2'—H2'···O1	0.88	2.42	3.025 (3)	126.
N3—H3···O1^ii^	0.88	2.34	3.129 (3)	149.

Symmetry codes: (i) −*x*+1, −*y*, −*z*+2; (ii) −*x*+2, −*y*, −*z*+2.
